# Association of New Perioperative Benzodiazepine Use With Persistent Benzodiazepine Use

**DOI:** 10.1001/jamanetworkopen.2021.12478

**Published:** 2021-06-03

**Authors:** Jason D. Wright, Jacob C. Cogan, Yongmei Huang, Ana I. Tergas, Caryn M. St. Clair, June Y. Hou, Fady Khoury-Collado, Allison Gockley, Melissa Accordino, Alexander Melamed, Dawn L. Hershman

**Affiliations:** 1 Columbia University College of Physicians and Surgeons, New York, New York; 2 Herbert Irving Comprehensive Cancer Center, New York, New York; 3 New York Presbyterian Hospital, New York; 4 Joseph L. Mailman School of Public Health, Columbia University, New York, New York

## Abstract

**Importance:**

Increased use of benzodiazepines has resulted in increasing rates of misuse and adverse effects associated with these drugs. Little is known about the initial exposure and source of benzodiazepines among those who use them persistently.

**Objective:**

To examine the frequency of use and persistent use of benzodiazepines among patients undergoing major and minor surgical procedures.

**Design, Setting, and Participants:**

This cohort study included 2 509 599 adult patients who underwent 1 of 11 common surgical procedures in the United States from 2009 to 2017 and were recorded in the MarketScan database. The rates of perioperative and persistent benzodiazepine use were examined in benzodiazepine-naive patients. Data analysis was conducted from July to November 2020.

**Main Outcomes and Measures:**

Receipt of a perioperative benzodiazepine prescription and persistent use (ie, fill of a second prescription 90-180 days after surgery) among those who received a benzodiazepine perioperatively.

**Results:**

Among 2 509 599 included patients, the mean (SD) age was 54.4 (15.3) years, and 1 596 137 (63.6%) were women. Perioperative benzodiazepine use was noted in 63 931 patients (2.6%). The median (interquartile range) benzodiazepine supply was 10 (5-23) days. Among benzodiazepine-naive patients prescribed a perioperative benzodiazepine, the rate of persistent benzodiazepine use was 19.5% (95% CI, 19.2%-19.8%). During the 90 to 180–day period after surgery, 7013 of 12 468 patients (56.2%) received 1 prescription for benzodiazepines while 5455 (43.8%) received 2 or more prescriptions. Among patients prescribed a benzodiazepine, persistent use was more common in Medicaid recipients (vs patients with commercial insurance: adjusted rate ratio [aRR], 1.29; 95% CI, 1.03-1.62), patients 70 years or older (vs those aged 40-49 years: aRR, 1.14; 95% CI, 1.05-1.23), in women (vs men: aRR, 1.10; 95% CI, 1.06-1.15), in patients with more medical comorbidities (eg, Elixhauser comorbidity score ≥3 vs 0: aRR, 1.11; 95% CI, 1.04-1.19), and in those with diagnoses of anxiety, depression, insomnia or substance use disorder (eg, with vs without anxiety: aRR, 1.43; 95% CI, 1.37-1.50).

**Conclusions and Relevance:**

In this study, a relatively small percentage of surgical patients were prescribed benzodiazepines in the perioperative period; however, 1 in 5 of these patients went on to persistent benzodiazepine use.

## Key Points

**Question:**

How frequently are benzodiazepines prescribed and used persistently among patients undergoing surgery?

**Findings:**

In this cohort study of more than 2.5 million patients who underwent 1 of 11 surgical procedures from 2009 to 2017, benzodiazepines were prescribed for 2.6% of patients. Among benzodiazepine-naive patients prescribed a perioperative benzodiazepine, the rate of persistent benzodiazepine use was 19.5%.

**Meaning:**

While a relatively small percentage of surgical patients in this cohort study were prescribed benzodiazepines in the perioperative period, 1 in 5 of these patients went on to persistent benzodiazepine use.

## Teaser

This cohort study examines the frequency of use and persistent use of benzodiazepines among patients undergoing major and minor surgical procedures.

## Introduction

Benzodiazepines are a class of drugs with sedative, anxiolytic, hypnotic, and anticonvulsant properties. These agents are commonly used for a variety of indications, including anxiety, insomnia, and panic disorders. In the United States, the use of benzodiazepines has increased substantially over the last 2 decades.^1,2,3^ From 1996 to 2013, prescriptions for benzodiazepines increased by 67%, from 8.1 million to 13.5 million prescriptions per year.^1^ A cross-sectional survey of adults in 2015 and 2016 reported that 13% of respondents had used benzodiazepines in the prior year.^3^

The increasing rate of benzodiazepine use has been accompanied by a rapid increase in adverse events.^1,4^ The rate of overdose deaths from benzodiazepines was 3.07 per 100 000 adults in 2010, a 4-fold increase from 1996.^1^ Benzodiazepines are also frequently involved in overdose deaths related to opioids and other synthetic narcotics.^1,5^ In addition, benzodiazepines have been implicated in numerous other adverse events, including emergency department visits for accidents, motor vehicle collisions, falls, and fractures.^2,6,7,8^ Benzodiazepines are currently the third most commonly misused illicit substance.^4^

The rise in benzodiazepine prescribing along with drug-related adverse events has led many observers to draw parallels with the opioid crisis.^2^ Current estimates suggest that 15% to 20% of benzodiazepines that are prescribed are misused.^3,9^ Among individuals misusing benzodiazepine, diversion appears to be common and illicit; synthetic benzodiazepines are becoming more prevalent.^4^ However, data on patient acquisition and maintenance of benzodiazepines remain limited. Similarly, how those who use benzodiazepines persistently first acquire these drugs and ultimately go on to prolonged use remains unclear. For opioids, exposure and persistent use of the drugs around the time of surgical procedures are common.^10,11^ Theoretically similar patterns may be seen for benzodiazepines, which may be prescribed during the perioperative period for anxiety, insomnia, or nausea. The objective of our study was to determine the prevalence of benzodiazepine prescribing in patients undergoing major and minor surgical procedures. Specifically, we examined the prescription of postoperative benzodiazepines and persistent use of benzodiazepines among those who received prescriptions.

## Methods

### Data Source and Study Cohort

The MarketScan Database (IBM Corp) was used for analysis. Annually, the MarketScan database captures claims from approximately 50 million enrollees with commercial insurance from 350 payers and 6 million Medicaid recipients from 12 states.^12^ The database includes billing data and claims for inpatient and outpatient services and pharmaceutical claims. The study was reported following the Strengthening the Reporting of Observational Studies in Epidemiology (STROBE) guidelines. Data were deidentified and deemed exempt from institutional review board approval and informed consent by the Columbia University institutional review board.

We selected patients aged 18 years or older who underwent 1 of 11 common surgical procedures performed from 2009 to 2017 in either the inpatient or outpatient setting. The procedures of interest included hip arthroplasty, knee arthroplasty, carpal tunnel surgery, appendectomy, cholecystectomy, thyroidectomy, colectomy, hemorrhoidectomy, hysterectomy, prostatectomy, pulmonary lobectomy, and cataract surgery (eTable 1 in the Supplement).

The cohort was limited to patients who had continuous health insurance, including prescription drug coverage, from 12 months before the procedure through 6 months after surgical intervention. To capture a cohort of patients who were benzodiazepine naive, we excluded patients who filled a prescription for a benzodiazepine during the period from 12 months to 31 days prior to surgery. As some patients may have filled a benzodiazepine prescription preoperatively or received it for preoperative indications, participants were included in the cohort if they filled a benzodiazepine prescription in the 30 days before surgery.^10,11,13^

### Benzodiazepine Pharmaceutical Claims

We examined outpatient pharmaceutical claims data to determine the type of benzodiazepine prescribed, duration of prescription in days, and number of refills. Prescription medications were identified by matching generic drug names and National Drug Codes to the MarketScan Micromedex Red Book, which provides specific drug dosing concentration (milligram/unit) and route of administration.^12^ For each prescription, we calculated the unit milligram of each benzodiazepine component, then converted that to oral lorazepam equivalents by using standard published conversions for the lorazepam equivalent conversion factor per milligram.^14^ The oral lorazepam equivalent dosage for each individual benzodiazepine prescription that was filled was calculated as the unit oral lorazepam equivalent exposure multiplied by the total quantity filled in the prescription.

Perioperative benzodiazepine use was defined as having filled at least 1 benzodiazepine prescription during 30 days before to 14 days after the procedure.^10^ New, persistent benzodiazepine use after surgery was defined as having filled 1 or more benzodiazpine prescriptions in the period from 90 to 180 days after surgery. This definition of persistent benzodiazepine use has been widely reported in the literature for persistent opioid use and represents a period when postoperative effects that may require a benzodiazepine have usually resolved.^10,15,16,17^

### Clinical and Demographic Characteristics

Clinical data included age at the time of surgery (ie, <40, 40-49, 50-59, 60-64, 65-69, or ≥70 years), insurance status (commercial insurance or Medicaid), self-reported gender (male or female), metropolitan statistical area (MSA; yes, no, or unknown), region (Northeast, North Central, South, West, or unknown), year of the index surgery, and hospital setting (outpatient or inpatient). Preoperative comorbidities were identified by *International Classification of Diseases, Ninth Revision *(*ICD-9*) and *Tenth Revision *(*ICD-10*) codes, and comorbidities were estimated using the Elixhauser Index.^18^

We analyzed the occurrence of underlying psychiatric diagnoses including insomnia, anxiety, depression, and substance use disorders (SUDs) during the 12-month period prior to surgery.^10^ We identified patients with coding for a diagnosis of cancer from 365 days prior to 180 days after surgery. Adjuvant chemotherapy and radiation after surgery were also captured. We examined opioid use prior to surgery, perioperative opioid use including receipt of a prescription for an opioid during the perioperative period (30 days before to 14 days after), and persistent opioid use, defined as having filled 1 or more opioid prescriptions in the period from 90 to 180 days after surgery in those patients who received a perioperative opioid prescription.^10,17,19,20^ We performed sensitivity analyses in which the classification of persistent benzodiazepine use required having filled 2 or more prescriptions in the period 90 to 180 days after surgery or receipt of a 30-day or longer supply of benzodiazepines.

### Statistical Analysis

Demographic and clinical characteristics are presented descriptively, and the rates of perioperative benzodiazepine use and new persistent benzodiazepine use were calculated for each covariate. The unadjusted association between the clinical and demographic characteristics and perioperative benzodiazepine use and persistent benzodiazepine use were compared using χ^2^ tests. Perioperative benzodiazepine prescription patterns (ie, total oral lorazepam equivalent dose, daily oral lorazepam equivalent dose dispensed, and days’ supply) and number of prescriptions are presented as medians with interquartile ranges (IQRs).

Multivariable log-linear models with Poisson distribution and log link function were developed to explore the factors associated with perioperative benzodiazepine use and new persistent benzodiazepine use. These models use a robust standard error estimator. In models of new persistent benzodiazepine use there was significant collinearity between initial total oral lorazepam equivalents, daily oral lorazepam equivalents, total days’ supply, prescription number, and benzodiazepine use within 30 days prior to surgery; separate models were estimated with inclusion of only 1 of the 5 variables. The initial total dose, daily dose, and total day supply were categorized into quartiles. The number of prescriptions was categorized as 1 or 2 or more. The results from log-linear models were reported as adjusted risk ratios (aRRs) with 95% CIs. All hypothesis tests were 2-sided, and statistical significance was set at *P* < .05. All analyses were conducted using SAS version 9.4 (SAS Institute).

## Results

**Figure 1.**
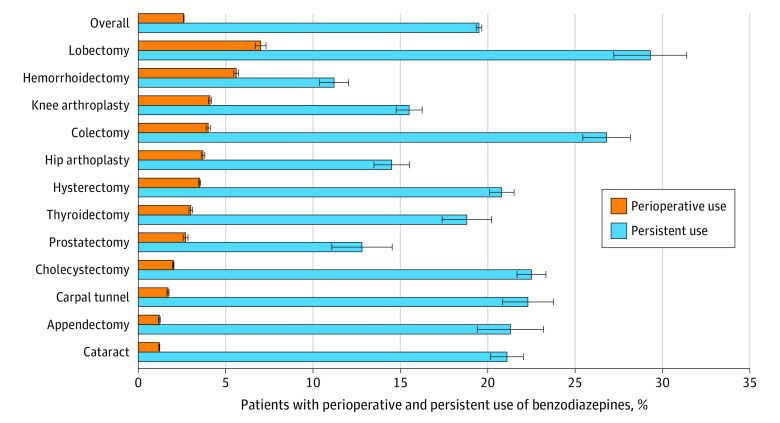
Perioperative and Persistent Benzodiazepine Use Stratified by Procedure Persistent use represents continued use only among those patients who were prescribed benzodiazepines in the perioperative period. Whiskers indicate 95% CIs.

**Figure 2.**
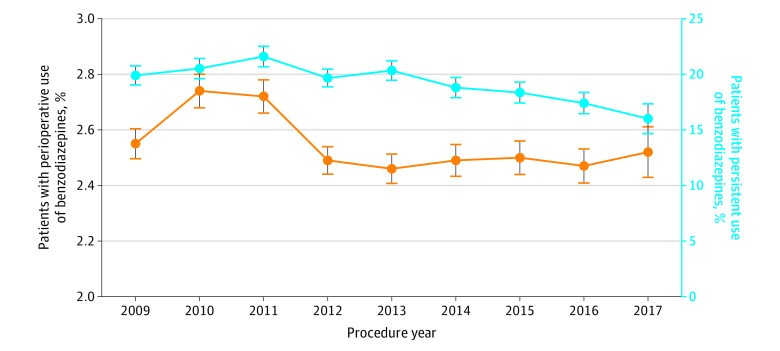
Use and Persistent Use of Perioperative Benzodiazepines by Year Orange line indicates use; blue line, persistent use; whiskers, 95% CIs.

A total of 2 509 599 patients were identified (Table 1). The mean (SD) age of the cohort was 54.4 (15.3) years, and 1 596 137 patients (63.6%) were women. Cataract surgery (618 575 [24.6%]) was the most commonly performed procedure, while lobectomy (26 061 [10.4%]) was received by the smallest subgroup of patients. Overall, perioperative benzodiazepine use was noted in 63 931 patients (2.6%; 95% CI, 2.53%-2.57%) (Figure 1). The most commonly used benzodiazepines were diazepam (20 661 [32.3%]) and alprazolam (18 794 [29.4%]) (eTable 2 in the Supplement). The median (IQR) days’ supply of benzodiazepine was 10 (5-23) days, and 35 088 patients (54.9%) filled a benzodiazepine prescription preoperatively (Table 2). The rate of perioperative benzodiazepine use was 2.5% (95% CI, 2.4%-2.6%) in 2009; it increased in 2010 and 2011 (2011 rate, 2.7%; 95% CI, 2.7%-2.8%) and then declined (2017 rate, 2.6%; 95% CI, 2.4%-2.6%) (Figure 2).

**Table 1.** Demographic and Clinical Factors Associated With Perioperative Benzodiazepines Use Among Benzodiazepine-Naive Patients

Characteristic	Patients, No. (%)			*P* value	aRR (95%CI)^b^
	All	Perioperative benzodiazepine use^a^			
		No	Yes		
All	2 509 599 (100.0)	2 445 668 (97.5)	63 931 (2.6)	NA	NA
Surgery type					
Hysterectomy	362 970 (14.5)	350 424 (96.5)	12 546 (3.5)	<.001	1.48 (1.44-1.52)^c^
Colectomy	100 649 (4.0)	96 590 (96.0)	4059 (4.0)		1.91 (1.83-1.99)^c^
Knee arthroplasty	221 340 (8.8)	212 335 (95.9)	9005 (4.1)		1.91 (1.85-1.98)^c^
Hip arthroplasty	125 477 (5.0)	120 823 (96.3)	4654 (3.7)		1.76 (1.69-1.83)^c^
Pulmonary lobectomy	26 061 (1.0)	24 231 (93.0)	1830 (7.0)		2.97 (2.82-3.14)^c^
Prostatectomy	52 564 (2.1)	51 129 (97.3)	1435 (2.7)		1.38 (1.30-1.47)^c^
Cholecystectomy	477 695 (19.0)	46 7931 (98.0)	9764 (2.0)		1 [Reference]
Thyroidectomy	97 241 (3.9)	94 323 (97.0)	2918 (3.0)		1.70 (1.63-1.77)^c^
Appendectomy	145 773 (5.8)	143 989 (98.8)	1784 (1.2)		0.66 (0.62-0.69)^c^
Cataract surgery	618 575 (24.7)	611 345 (98.8)	7230 (1.2)		1.51 (1.45-1.57)^c^
Hemorrhoidectomy	98 765 (3.9)	93 229 (94.4)	5536 (5.6)		3.78 (3.65-3.91)^c^
Carpal tunnel surgery	182 489 (7.3)	179 319 (98.3)	3170 (1.7)		0.91 (0.87-0.95)^c^
Age at surgery, y					
<40	430 513 (17.2)	418 996 (97.3)	11 517 (2.7)	<.001	0.97 (0.95-1.00)^d^
40-49	456 577 (18.2)	442 795 (97.0)	13 782 (3.0)		1 [Reference]
50-59	649 498 (25.9)	630 677 (97.1)	18 821 (2.9)		1.00 (0.98-1.03)
60-64	426 835 (17.0)	415 995 (97.5)	10 840 (2.5)		0.96 (0.94-0.99)^d^
65-69	154 247 (6.2)	150 898 (97.8)	3349 (2.2)		0.92 (0.89-0.96)^d^
≥70	391 929 (15.6)	386 307 (98.6)	5622 (1.4)		0.74 (0.72-0.77)^c^
Health insurance					
Commercial	2 344 978 (93.4)	2 286 856 (97.5)	58 122 (2.5)	<.001	1 [Reference]
Medicaid	164 621 (6.6)	158 812 (96.5)	5809 (3.5)		1.15 (1.05-1.26)^d^
Gender					
Male	913 462 (36.4)	893 771 (97.8)	19 691 (2.2)	<.001	1 [Reference]
Female	1 596 137 (63.6)	1 551 897 (97.2)	44 240 (2.8)		1.22 (1.19-1.24)^c^
MSA					
MSA	1895 669 (75.5)	1 848 384 (97.5)	47 285 (2.5)	<.001	1 [Reference]
Non-MSA	407 118 (16.2)	397 204 (97.6)	9914 (2.4)		1.01 (0.99-1.03)
Unknown	206 812 (8.2)	200 080 (96.7)	6732 (3.3)		0.97 (0.88-1.07)
Region					
Northeast	399 734 (15.9)	390 426 (97.7)	9308 (2.3)	<.001	1 [Reference]
North Central	620 249 (24.7)	605 781 (97.7)	14 468 (2.3)		0.99 (0.97-1.02)
South	937 602 (37.4)	914 378 (97.5)	23 224 (2.5)		1.00 (0.97-1.02)
West	366 418 (14.6)	355 783 (97.1)	10 635 (2.9)		1.17 (1.13-1.20)^c^
Unknown	185 596 (7.4)	179 300 (96.6)	6296 (3.4)		0.92 (0.81-1.05)
Year of index procedure					
2009	326 843 (13.0)	318 524 (97.5)	8319 (2.6)	<.001	1 [Reference]
2010	277 922 (11.1)	270 300 (97.3)	7622 (2.7)		0.95 (0.93-0.99)^d^
2011	284 308 (11.3)	276 576 (97.3)	7732 (2.7)		0.95 (0.92-0.98)^d^
2012	383 290 (15.3)	373 765 (97.5)	9525 (2.5)		0.97 (0.94-1.00)
2013	331 875 (13.2)	323 703 (97.5)	8172 (2.5)		0.93 (0.90-0.96)^c^
2014	285 885 (11.4)	278 758 (97.5)	7127 (2.5)		0.92 (0.89-0.95)^c^
2015	259 412 (10.3)	252 928 (97.5)	6484 (2.5)		0.89 (0.86-0.92)^c^
2016	246 149 (9.8)	240 068 (97.5)	6081 (2.5)		0.86 (0.83-0.89)^c^
2017	113 915 (4.5)	111 046 (97.5)	2869 (2.5)		0.87 (0.83-0.91)^c^
Hospital setting					
Outpatient	1668 317 (66.5)	163 4501 (98.0)	33 816 (2.0)	<.001	1 [Reference]
Inpatient	841 282 (33.5)	811 167 (96.4)	30 115 (3.6)		1.19 (1.17-1.22)^c^
Elixhauser comorbidity score					
0	1631 339 (65.0)	1 590 906 (97.5)	40 433 (2.5)	<.001	1 [Reference]
1	514 179 (20.5)	500 407 (97.3)	13 772 (2.7)		0.98 (0.96-1.00)
2	226 687 (9.0)	220 801 (97.4)	5886 (2.6)		0.92 (0.90-0.95)^c^
≥3	137 394 (5.5)	133 554 (97.2)	3840 (2.8)		0.88 (0.85-0.91)^c^
Insomnia					
No	2 454 156 (97.8)	2393 160 (97.5)	60 996 (2.5)	<.001	1 [Reference]
Yes	55 443 (2.2)	52 508 (94.7)	2935 (5.3)		1.52 (1.46-1.58)^c^
Anxiety					
No	2 346 017 (93.5)	2 293 134 (97.8)	52 883 (2.3)	<.001	1 [Reference]
Yes	163 582 (6.5)	152 534 (93.3)	11 048 (6.8)		2.63 (2.57-2.69)^c^
Depression					
No	2 340 417 (93.3)	2 283 489 (97.6)	56 928 (2.4)	<.001	1 [Reference]
Yes	169 182 (6.7)	162 179 (95.9)	7003 (4.1)		1.08 (1.05-1.11)^c^
Substance use disorder					
No	2 333 673 (93.0)	2 276 564 (97.6)	57 109 (2.5)	<.001	1 [Reference]
Yes	175 926 (7.0)	169 104 (96.1)	6822 (3.9)		1.16 (1.13-1.19)^c^
Malignant diseases					
No	1 954 556 (77.9)	1 908 650 (97.7)	45 906 (2.4)	<.001	1 [Reference]
Yes	555 043 (22.1)	537 018 (96.8)	18 025 (3.3)		1.31 (1.28-1.34)^c^
Presurgery opioid use					
No	1 582 491 (63.1)	1 549 001 (97.9)	33 490 (2.1)	<.001	1 [Reference]
Yes	927 108 (36.9)	896 667 (96.7)	30441 (3.3)		1.21 (1.19-1.23)^c^
Perioperative opioid use					
No	937 308 (37.4)	926 821 (98.9)	10 487 (1.1)	<.001	1 [Reference]
Yes	1 572 291 (62.7)	1 518 847 (96.6)	53 444 (3.4)		2.66 (2.60-2.74)^c^

Abbreviations: aRR, adjusted rate ratio; MSA, Metropolitan statistical area; NA, not applicable.

^a^ Percentages are row percentages.

^b^ Log-linear model with Poisson distribution and log link function.

^c^ *P* < .001.

^d^ *P* < .05.

**Table 2.** Perioperative and Persistent Benzodiazepine Prescription Patterns Stratified by Surgery Type

Surgery	Patients, No. (%)	Median (IQR)					Patients receiving ≥2 benzodiazepine prescriptions, No. (%)
		Total oral lorazepam equivalents, mg		Daily oral lorazepam equivalents, mg	Initial total day supply, d		
**Perioperative benzodiazepine prescription filled**							
All	63 931 (100.0)		15.0 (6.0-30.0)	1.5 (01.0-2.0)		10 (5-23)	6989 (10.9)
Hysterectomy	12 546 (19.6)		15.0 (6.0-30.0)	1.5 (1.0-2.5)		10 (5-20)	1323 (10.6)
Colectomy	4059 (6.3)		17.5 (10.0-30.0)	1.5 (1.0-2.5)		10 (7-26)	469 (11.6)
Knee arthroplasty	9005 (14.1)		15.0 (10.0-30.0)	1.5 (1.0-2.0)		10 (7-21)	947 (10.5)
Hip arthroplasty	4654 (7.3)		15.0 (10.0-30.0)	1.5 (1.0-2.0)		10 (7-20)	441 (9.5)
Pulmonary lobectomy	1830 (2.9)		21.0 (10.0-40.0)	1.5 (1.0-2.3)		15 (7-30)	324 (17.7)
Prostatectomy	1435 (2.2)		15.0 (4.0-30.0)	1.5 (1.0-2.0)		10 (3-20)	147 (10.2)
Cholecystectomy	9764 (15.3)		15.0 (5.0-30.0)	1.5 (0.8-2.0)		10 (4-30)	1015 (10.4)
Thyroidectomy	2918 (4.6)		15.0 (4.0-30.0)	1.4 (1.0-2.0)		10 (3-30)	320 (11)
Appendectomy	1784 (2.8)		15.0 (5.0-30.0)	1.5 (1.0-2.0)		10 (4-30)	145 (8.1)
Cataract	7230 (11.3)		12.5 (2.0-30.0)	1.0 (0.8-2.0)		10 (2-30)	669 (9.3)
Hemorrhoidectomy	5536 (8.7)		15.0 (10.0-30.0)	1.5 (1.0-2.1)		10 (5-15)	883 (16)
Carpal tunnel surgery	3170 (5.0)		14.0 (2.0-30.0)	1.3 (1.0-2.0)		8 (1-30)	306 (9.7)
**Postoperative benzodiazepine prescription filled within 90-180 d after surgery**							
All	12 468 (100.0)		45.0 (20.0-100.0)	1.5 (1.0-2.7)		30 (15-60)	5455 (43.8)
Hysterectomy	2609 (20.9)		45.0 (20.0-95.0)	1.9 (1.0-3.0)		30 (15-60)	1182 (45.3)
Colectomy	1086 (8.7)		60.0 (30.0-120.0)	1.9 (1.0-3.0)		30 (15-60)	530 (48.8)
Knee arthroplasty	1399 (11.2)		36.0 (20.0-90.0)	1.5 (1.0-2.0)		30 (15-60)	531 (38)
Hip arthroplasty	676 (5.4)		30.0 (15.0-90.0)	1.5 (1.0-2.1)		30 (13-60)	252 (37.3)
Pulmonary lobectomy	536 (4.3)		60.0 (30.0-120.0)	1.8 (1.0-3.0)		30 (22.5-60)	287 (53.5)
Prostatectomy	183 (1.5)		45.0 (30.0-90.0)	1.5 (1.0-3.0)		30 (20-60)	75 (41)
Cholecystectomy	2195 (17.6)		60.0 (25.0-120.0)	1.5 (1.0-2.7)		30 (20-60)	1064 (48.5)
Thyroidectomy	547 (4.4)		37.5 (20.0-90.0)	1.5 (1.0-2.0)		30 (15-60)	199 (36.4)
Appendectomy	380 (3.0)		45.0 (20.0-120.0)	1.8 (1.0-3.0)		30 (16.5-60)	172 (45.3)
Cataract	1527 (12.2)		45.0 (20.0-90.0)	1.1 (1.0-2.0)		30 (24-60)	587 (38.4)
Hemorrhoidectomy	622 (5.0)		32.5 (15.0-90.0)	1.5 (1.0-2.5)		30 (10-60)	251 (40.4)
Carpal tunnel surgery	708 (5.7)		45.0 (18.0-120.0)	1.5 (1.0-2.1)		30 (20-73)	325 (45.9)

Abbreviation: IQR, interquartile range.

The highest rate of perioperative benzodiazepine use was among patients who underwent lobectomy (1830 of 26 061 [7.0%]) followed by hemorrhoidectomy (5536 of 98 765 [5.6%]), while the lowest rate of use was seen in those who underwent cataract surgery (7230 of 618 575 [1.2%]), appendectomy (1784 of 145 773 [1.2%]), or carpal tunnel surgery (3170 of 182 489 [1.7%]) (Table 1 and Figure 1). Medicaid recipients were 15% more likely to receive a perioperative benzodiazepine prescription than commercially insured patients (5809 of 164 621 [3.5%] vs 58 122 of 2 344 978 [2.5%]; aRR, 1.15; 95% CI, 1.05-1.26). Use of benzodiazepines was lower with older age, among patients who underwent surgery more recently, and among men. Patients with a diagnosis of anxiety, depression, insomnia, SUD, or cancer were more likely to receive a benzodiazepine. Similarly, patients who had used opioids prior to the perioperative period or during the perioperative period were more likely to receive a benzodiazepine prescription.

Among benzodiazepine-naive patients prescribed a perioperative benzodiazepine, the rate of persistent benzodiazepine use was 19.5% (95% CI, 19.2%-19.8%). The rate of persistent benzodiazepine use was 19.9% (95% CI, 19.0%-20.8%) in 2009, increased slightly through 2011 (21.6%; 95% CI, 20.7%-22.5%) and then decreased (2017 rate, 16.0%; 95% CI, 14.7%-17.3%) (Figure 2). During the 90 to 180–day period after surgery, 7013 of 12 648 individuals (56.2%) with persistent benzodiazepine use received 1 prescription for benzodiazepines, while 5455 (43.8%) received 2 or more prescriptions (Table 2). Alprazolam was the most commonly used benzodiazepine (4619 [37.5%]) during this period, followed by lorazepam (3383 [27.4%]) (eTable 2 in the Supplement). The median (IQR) days’ supply of benzodiazepine during this period was 30 (15-60) days.

Medicaid recipients were 29% more likely to persistently use benzodiazepines than participants with commercial insurance (1547 of 5809 [26.6%] vs 10 921 of 58 122 [18.8%]; aRR, 1.29; 95% CI, 1.03-1.62) (Table 3). Persistent use of benzodiazepines was most common after lobectomy (536 of 1830 [29.3%]), colectomy (1086 of 4059 [26.8%]), and cholecystectomy (2195 of 9764 [22.5%]). Persistent benzodiazepine use was more common in women than in men (9143 of 44 240 [20.7%] vs 3325 of 19 691 [16.9%]; aRR, 95% CI, 1.10; 1.06-1.15), among older patients (≥70 years vs 40-49 years: 1147 of 5622 [20.4%] vs 2636 of 13 782 [21.6%]; aRR, 1.14; 95% CI, 1.05-1.23), among residents in the South compared with the Northeast (4683 of 23 224 [20.2%] vs 1650 of 9308 [17.7%]; aRR, 1.16; 95% CI, 1.10-1.23), in patients with more medical comorbidities (eg, with Elixhauser comorbidity score ≥3 vs 0: 1044 of 3840 [27.2%] vs 7229 of 40 433 [17.9%]; aRR, 1.11; 95% CI, 1.04-1.19), and for those who underwent an inpatient procedure compared with outpatient procedure (6015 of 30 115 [20.0%] vs 6453 of 33 816 [19.1%]; aRR, 1.07; 95% CI, 1.01-1.12). Patients with a diagnosis of cancer and those who received chemotherapy or radiation were also more likely to persistently use benzodiazepines. Similarly, diagnoses of anxiety, depression, insomnia, and SUD were all associated with an increased rate of persistent benzodiazepine use (eg, anxiety: 3168 of 11 048 [28.7%] vs 9300 of 52 883 [17.6%]; aRR, 1.43; 95% CI, 1.37-1.50).

**Table 3.** Demographic and Clinical Factors Associated With Persistent Benzodiazepines Use

Characteristic	Patients, No. (%)			*P* value	aRR (95%CI)^b^
	All	Persistent use^a^			
		No	Yes		
**Patient characteristics**					
All	63 931 (100)	51 463 (80.5)	12 468 (19.5)	NA	NA
Surgery type					
Hysterectomy	12 546 (19.6)	9937 (79.2)	2609 (20.8)	<.001	0.88 (0.83-0.93)^c^
Colectomy	4059 (6.4)	2973 (73.2)	1086 (26.8)		0.96 (0.88-1.04)
Knee arthroplasty	9005 (14.1)	7606 (84.5)	1399 (15.5)		0.73 (0.67-0.79)^c^
Hip arthroplasty	4654 (7.3)	3978 (85.5)	676 (14.5)		0.69 (0.62-0.76)^c^
Pulmonary lobectomy	1830 (2.9)	1294 (70.7)	536 (29.3)		0.93 (0.84-1.03)
Prostatectomy	1435 (2.2)	1252 (87.3)	183 (12.8)		0.62 (0.53-0.73)^c^
Cholecystectomy	9764 (15.3)	7569 (77.5)	2195 (22.5)		1 [Reference]
Thyroidectomy	2918 (4.6)	2371 (81.3)	547 (18.7)		0.84 (0.77-0.93)^d^
Appendectomy	1784 (2.8)	1404 (78.7)	380 (21.3)		1.00 (0.89-1.11)
Cataract surgery	7230 (11.3)	5703 (78.9)	1527 (21.1)		1.03 (0.95-1.12)
Hemorrhoidectomy	5536 (8.7)	4914 (88.8)	622 (11.2)		0.62 (0.56-0.68)^c^
Carpal tunnel surgery	3170 (5.0)	2462 (77.7)	708 (22.3)		1.01 (0.93-1.10)
Age at surgery, y					
<40	11 517 (18.0)	9127 (79.3)	2390 (20.7)	.01	0.99 (0.93-1.04)
40-49	13 782 (21.6)	11 146 (80.9)	2636 (19.1)		1 [Reference]
50-59	18 821 (29.4)	15 233 (80.9)	3588 (19.1)		1.01 (0.96-1.07)
60-64	10 840 (17.0)	8743 (80.7)	2097 (19.3)		1.05 (0.99-1.12)
65-69	3349 (5.2)	2739 (81.8)	610 (18.2)		1.03 (0.94-1.14)
≥70	5622 (8.8)	4475 (79.6)	1147 (20.4)		1.14 (1.05-1.23)^d^
Health insurance					
Commercial	58 122 (90.9)	47 201 (81.2)	10 921 (18.8)	<.001	1 [Reference]
Medicaid	5809 (9.1)	4262 (73.4)	1547 (26.6)		1.29 (1.03-1.62)^d^
Gender					
Male	19 691 (30.8)	16 366 (83.1)	3325 (16.9)	<.001	1 [Reference]
Female	44 240 (69.2)	35 097 (79.3)	9143 (20.7)		1.10 (1.06-1.15)^c^
MSA					
MSA	9914 (15.5)	8039 (81.1)	1875 (18.9)	<.001	0.97 (0.92-1.02)
Non-MSA	47 285 (74.0)	38 396 (81.2)	8889 (18.8)		1 [Reference]
Unknown	6732 (10.5)	5028 (74.7)	1704 (25.3)		1.10 (0.86-1.39)
Region					
Northeast	9308 (14.6)	7658 (82.3)	1650 (17.7)	<.001	1 [Reference]
North Central	14 468 (22.6)	11 810 (81.6)	2658 (18.4)		1.05 (0.99-1.12)
South	23 224 (36.3)	18 541 (79.8)	4683 (20.2)		1.16 (1.10-1.23)^c^
West	10 635 (16.6)	8787 (82.6)	1848 (17.4)		1.01 (0.94-1.08)
Unknown	6296 (9.9)	4667 (74.1)	1629 (25.9)		0.86 (0.62-1.18)
Year of index procedure					
2009	8319 (13.0)	6664 (80.1)	1655 (19.9)	<.001	1 [Reference]
2010	7622 (11.9)	6059 (79.5)	1563 (20.5)		0.99 (0.92-1.06)
2011	7732 (12.1)	6063 (78.4)	1669 (21.6)		1.05 (0.98-1.12)
2012	9525 (14.9)	7652 (80.3)	1873 (19.7)		0.93 (0.87-1.00)^d^
2013	8172 (12.8)	6511 (79.7)	1661 (20.3)		0.94 (0.87-1.00)
2014	7127 (11.2)	5787 (81.2)	1340 (18.8)		0.85 (0.79-0.91)^c^
2015	6484 (10.1)	5294 (81.7)	1190 (18.3)		0.79 (0.73-0.85)^c^
2016	6081 (9.5)	5023 (82.6)	1058 (17.4)		0.74 (0.68-0.80)^c^
2017	2869 (4.5)	2410 (84.0)	459 (16.0)		0.67 (0.60-0.75)^c^
Hospital setting					
Outpatient	33 816 (52.9)	27 363 (80.9)	6453 (19.1)	.01	1 [Reference]
Inpatient	30 115 (47.1)	24 100 (80.0)	6015 (20.0)		1.07 (1.01-1.12)^d^
Elixhauser comorbidity score					
0	40 433 (63.2)	33 204 (82.12)	7229 (17.9)	<.001	1 [Reference]
1	13 772 (21.5)	10 941 (79.4)	2831 (20.6)		1.03 (0.99-1.08)
1	5886 (9.2)	4522 (76.8)	1364 (23.2)		1.09 (1.03-1.16)^d^
≥3	3840 (6.0)	2796 (72.8)	1044 (27.2)		1.11 (1.04-1.19)^d^
Insomnia					
No	60 996 (95.4)	49 461 (81.1)	11 535 (18.9)	<.001	1 [Reference]
Yes	2935 (4.6)	2002 (68.2)	933 (31.8)		1.41 (1.32-1.51)^c^
Anxiety					
No	52 883 (82.7)	43 583 (82.4)	9300 (17.6)	<.001	1 [Reference]
Yes	11 048 (17.3)	7880 (71.3)	3168 (28.7)		1.43 (1.37-1.50)^c^
Depression					
No	56 928 (89.1)	46 493 (81.7)	10 435 (18.3)	<.001	1 [Reference]
Yes	7003 (10.9)	4970 (71.0)	2033 (29.0)		1.24 (1.18-1.31)^c^
Substance use disorders					
No	57 109 (89.3)	46 525 (81.5)	10 584 (18.5)	<.001	1 [Reference]
Yes	6822 (10.7)	4938 (72.4)	1884 (27.6)		1.18 (1.11-1.24)^c^
Malignant diseases					
No	45 906 (71.8)	37 656 (82.0)	8250 (18.0)	<.001	1 [Reference]
Yes	18 025 (28.2)	13 807 (76.6)	4218 (23.4)		1.06 (1.01-1.11)^d^
Chemotherapy					
No	59 549 (93.2)	48 766 (81.9)	10 783 (18.1)	<.001	1 [Reference]
Yes	4382 (6.8)	2697 (61.5)	1685 (38.5)		1.62 (1.51-1.74)^c^
Radiation					
No	61 555 (96.3)	50 045 (81.3)	11 510 (18.7)	<.001	1 [Reference]
Yes	2376 (3.7)	1418 (59.7)	958 (40.3)		1.31 (1.20-1.43)^c^
Presurgery opioid use					
No	33 490 (52.4)	27 863 (83.2)	5627 (16.8)	<.001	1 [Reference]
Yes	30 441 (47.6)	23 600 (77.5)	6841 (22.5)		1.47 (1.41-1.53)^c^
Perioperative opioid use					
No	10 487 (16.4)	8434 (80.4)	2053 (19.6)	.83	1 [Reference]
Yes	53 444 (83.6)	43 029 (80.5)	10 415 (19.5)		0.91 (0.85-0.96)^d^
Persistent opioid use					
No	59 436 (93.0)	48 436 (81.5)	11 000 (18.5)	<.001	1 [Reference]
Yes	4495 (7.0)	3027 (67.3)	1468 (32.7)		2.09 (1.96-2.22)^c^
**Initial perioperative benzodiazepine use**					
Total oral lorazepam equivalents, mg^e^					
<6.0	14 539 (22.7)	13 309 (91.5)	1230 (8.5)	<.001	1 [Reference]
6.0-14.9	11 537 (18.1)	10 184 (88.3)	1353 (11.7)		1.51 (1.39-1.63)^c^
15.0-29.9	17 723 (27.7)	14 489 (81.8)	3234 (18.3)		2.32 (2.17-2.48)^c^
≥30.0	20 132 (31.5)	13 481 (67.0)	6651 (33.0)		3.66 (3.44-3.90)^c^
Daily oral lorazepam equivalents, mg^e^					
<1.00	14 390 (22.5)	12 086 (84.0)	2304 (16.0)	<.001	1 [Reference]
1.00-1.49	14 676 (23.0)	11 782 (80.3)	2894 (19.7)		1.20 (1.13-1.27)^c^
1.50-1.99	11 556 (18.1)	9496 (82.2)	2060 (17.8)		1.14 (1.08-1.21)^c^
≥2.00	23 309 (36.5)	18 099 (77.7)	5210 (22.4)		1.35 (1.28-1.41)^c^
Total days’ supply^e^					
<5	14 189 (22.2)	12 979 (91.5)	1208 (8.5)	<.001	1 [Reference]
5-9	12 553 (19.6)	10 955 (87.3)	1598 (12.7)		1.60 (1.48-1.72)^c^
10-22	21 039 (32.9)	17 171 (81.6)	3870 (18.4)		2.22 (2.08-2.38)^c^
≥23	16 150 (25.3)	10 358 (64.1)	5792 (35.9)		3.88 (3.64-4.13)^c^
Prescriptions, No.^e^					
1	56 942 (89.1)	47 023 (82.6)	9919 (17.4)	<.001	1 [Reference]
≥2	6989 (10.9)	4440 (63.5)	2549 (36.5)		1.87 (1.79-1.96)^c^
Presurgery use within 30 d^e^					
No	28 843 (45.1)	24 251 (84.1)	4592 (15.9)	<.001	1 [Reference]
Yes	35 088 (54.9)	27 212 (77.6)	7876 (22.4)		1.23 (1.18-1.27)^c^

Abbreviations: aRR, adjusted rate ratio; MSA, metropolitan statistical area; NA, not applicable.

^a^ Percentages are row percentages.

^b^ Log-linear models with Poisson distribution and log link function.

^c^ *P* < .001.

^d^ *P* < .05.

^e^ Due to the multicollinearity between perioperative benzodiazepine prescription measurements, the association between perioperative benzodiazepine use and persistent use were examined in separate models. Each model adjusted for patients’ demographic and clinical factors.

Patients who initially received a higher total dose of benzodiazepines (eg, ≥30 mg vs <6 mg: 6651 of 20 132 [33.0%] vs 1230 of 14 539 [8.5%]; aRR, 3.66; 95% CI, 3.44-3.90), a higher daily dose of benzodiazepines (eg, ≥2.0 mg/d vs <1.0 mg/d: 5210 of 23 309 [22.4%] vs 2304 of 14 390 [16.0%]; aRR, 1.35; 95% CI, 1.28-1.41), and a longer duration prescription of benzodiazepines (eg, ≥23 days vs <5 days: 5792 of 16 150 [35.9%] vs 1208 of 14 189 [8.5%]; 3.88; 95% CI, 3.64-4.13) were more likely to persistently use benzodiazepines. Similarly, patients who received a benzodiazepine prescription in the 30-day period prior to surgery and those who received more than 1 benzodiazepine prescription perioperatively more commonly went on to persistent benzodiazepine use. Persistent benzodiazepine use was also more common among patients who used opioids persistently (Table 3).

These findings were robust in a series of sensitivity analyses. When persistent benzodiazepine use was defined as receipt of a 30-day or longer supply of benzodiazepines in the period from 90 to 180 days after surgery, the rate of persistent use was 12.9% (8257 patients) (eTable 3 in the Supplement). Similarly, defining persistent benzodiazepine use as the fill of 2 or more prescriptions 90 to 180 days after surgery was associated with a persistence rate of 8.5% (5455 patients) (eTable 4 in the Supplement). In both of these analyses, the factors associated with persistent use were similar.

## Discussion

These data suggest that while a relatively small percentage of surgical patients are prescribed benzodiazepines in the perioperative period, 1 in 5 of these patients will go on to persistent benzodiazepine use. In addition to clinical characteristics, patterns of benzodiazepine prescribing are strongly associated with persistent use and are a potentially modifiable factor to reduce persistent benzodiazepine use.

Population-level data suggest that benzodiazepine misuse is becoming an increasingly important public health challenge.^1,2,3^ From 1996 to 2013, the death rate from overdose involving benzodiazepine increased by more than 400%.^1,4^ While the death rate from benzodiazepine overdose plateaued after 2010, perhaps related to efforts focused on opioid safety, death rates have continued to increase for older adults as well as Black and Hispanic populations. Over the same time period, the quantity of benzodiazepines filled per prescription more than doubled.^1^ Among patients newly exposed to benzodiazepines at the time of surgery, we noted a substantial risk of continued use after the acute perioperative period. These findings mimic what has been described for opioid use and suggest that surgical procedures may serve as an important exposure for persistent benzodiazepine use.^10,19,21^

Prior studies have shown that the potential for misuse is high among patients exposed to benzodiazepines.^4,22,23,24,25,26^ One report suggested that prescription of an anxiety medication was associated with a 60% to 90% increased risk of nonmedical use of these drugs.^23^ The high rate of benzodiazepine misuse among those exposed to the drug may reflect ease of access to the medications, a greater abuse potential among those who initially use benzodiazepines, and purposeful seeking of benzodiazepine prescriptions among those who intend to misuse the drugs.^4^ History of SUD, younger age at receipt of a benzodiazepine prescription, longer duration of use, and a higher frequency of prescription use have all been associated with benzodiazepine misuse.^4,22,23,24,25,26^ In line with the high potential for misuse, we noted that a remarkable 20% of benzodiazepine-naive patients who received a prescription in the perioperative period went on to persistently use the drugs.

While misused benzodiazepines may be obtained from a variety of sources, diversion from family members and friends is the most common origin of the drugs.^4,27,28,29^ One survey found that nearly two-thirds of misused tranquilizers were obtained from either friends or family members and that most of these drugs were originally ascertained from a prescription.^4,27^ These findings heighten the concern that perioperative benzodiazepines may be an important source of diverted drugs.

Coingestion of benzodiazepines with other drugs, particularly opioids, is common.^30,31^ An ecologic time series study found that nearly 10% of opioid recipients were dispensed a concomitant benzodiazepine in 2014.^30^ Nearly half of these patients received the 2 prescriptions from the same health care practitioner on the same day.^30^ Coingestion of benzodiazepines and opioids is particularly problematic, as benzodiazepines enhance the effects of opioids and increase the risk of overdose and death.^4,31^ The increasing rate of benzodiazepines parallels that of the opioid epidemic in that short-term prescription of these agents for medical indications appears to lead to persistent use of the drugs. Our data suggest that perioperative benzodiazepine use is an important source for prolonged and persistent benzodiazepine use. Initial and persistent opioid use were also significantly associated with persistent benzodiazepine use. Encouragingly, the rate of initial and persistent benzodiazepine use appears to be decreasing.

### Limitations

We acknowledge a number of important limitations in the current analysis. First, using observational data, we are unable to determine appropriateness of the benzodiazepine prescriptions distributed. While we recognize that there are many appropriate uses for benzodiazepines, a priori the goal of the analysis was to simply document persistent use of these drugs regardless of medical indication. Second, while we report receipt of benzodiazepine prescriptions, we have no way to ascertain actual benzodiazepine consumption by patients through the pharmacy claims data we used. Third, our data were drawn from commercially insured patients and a subset of Medicaid recipients. As such, these data may not be generalizable to other populations, including Medicare beneficiaries. Fourth, we cannot identify the source of benzodiazepines, as we lack data on the specific health care practitioner who provided the prescriptions.

## Conclusions

This study found that although a small percentage of surgical patients receive prescriptions for benzodiazepines, 1 in 5 of them go on to use them persistently. From a policy perspective, these data have important implications. To date, misuse of benzodiazepines has received much less attention than misuse of opioids. Our findings suggest that efforts are needed to encourage the judicious use of these drugs after surgery. Benzodiazepines should only be used in patients with a clear indication, and attempts should be made to limit the quantity and duration of use. Similar efforts have been successfully used to reduce perioperative opioid prescription use. When appropriate, alternative medications with less potential for misuse should be strongly considered. Finally, raising awareness among patients, health care practitioners, and policy makers and implementing pragmatic strategies to limit use may help to curb misuse of benzodiazepine associated with perioperative prescribing.

## Notes

Supplement. **eTable 1.** *ICD* and *CPT* Codes for Procedures and Disorders and Drug Names Included for Identifying Benzodiazepine Prescriptions **eTable 2.** Individual Benzodiazepine Drugs Used Perioperatively and Persistently **eTable 3.** Factors Associated With at Least 30 Days’ Benzodiazepine Supply During 90 to 180 Days After Operation **eTable 4.** Factors Associated With 2 or More Benzodiazepine Prescriptions Filled 90 to 180 Days After Surgery
