# Supplementary material for: Association of New Perioperative Benzodiazepine Use With Persistent Benzodiazepine Use
Source: JAMA Netw Open. 2021 Jun 3;4(6):e2112478. doi: 10.1001/jamanetworkopen.2021.12478 (PMC8176328; doi:10.1001/jamanetworkopen.2021.12478)
Supplement: Supplement. — eTable 1. ICD and CPT Codes for Procedures and Disorders and Drug Names Included for Identifying Benzodiazepine Prescriptions eTable 2. Individual Benzodiazepine Drugs Used Perioperatively and Persistently eTable 3. Factors Associated With at Least 30 Days’ Benzodiazepine Supply During 90 to 180 Days After Operation eTable 4. Factors Associated With 2 or More Benzodiazepine Prescriptions Filled 90 to 180 Days After Surgery [file jamanetwopen-e2112478-s001.pdf]

Supplemental Online Content

Wright JD, Cogan JC, Huang Y, et al. Association of new perioperative benzodiazepine use with persistent benzodiazepine use. *JAMA Netw Open*. 2021;4(6):e2112478. doi:10.1001/jamanetworkopen.2021.12478

eTable 1. *ICD* and *CPT* Codes for Procedures and Disorders and Drug Names Included for Identifying Benzodiazepine Prescriptions
eTable 2. Individual Benzodiazepine Drugs Used Perioperatively and Persistently
eTable 3. Factors Associated With at Least 30 Days' Benzodiazepine Supply During 90 to 180 Days After Operation
eTable 4. Factors Associated With 2 or More Benzodiazepine Prescriptions Filled 90 to 180 Days After Surgery

This supplemental material has been provided by the authors to give readers additional information about their work.

© 2021 Wright JD et al. *JAMA Network Open.*

eTable 1. *ICD* and *CPT* Codes for Procedures and Disorders and Drug Names Included for Identifying Benzodiazepine Prescriptions

**Procedure/ Diagnosis**	**Codes**
Benzodiazepine	Alprazolam, Bromazepam, Chlordiazepoxide, Clobazam, Clonazepam, Clorazepate, Diazepam, Estazolam Flurazepam, Lorazepam, Midazolam, Nitrazepam, Oxazepam, Quazepam, Remimazolam, Temazepam, Triazolam, Halazepam, Prazepam
Hysterectomy	ICD9: 68.3, 68.39, 68.4, 68.49, 68.6, 68.69, 68.9, 68.31, 68.41, 68.51, 68.61, 68.71, 68.5, 68.59, 68.7, 68.79<br>ICD10: 0UT90b, 0UT94b, 0UT98b, 0UT9Fb, 0UT97b<br>CPT: 58150,58152,58180,58200,58210,58240,58950,58951,58953,58956,58594, 58541,58542,58543, 58544,58548, 58550,58552,58553,58554,58570,58571,58572,58573, 58260,58262,58263,58267,58270,58275,58280,58285,58290, 58291,58292,58293,58294
Thyroidectomy	ICD9: 06.2,06.98,06.9,06.3,06.39, 06.4,06.5,06.51,06.52<br>ICD10: 0GBHb, 0GBGb, OGBJb, 0GCGb, 0GCHb, 0GTGb, 0GTHb, 0GCKb, 0GTJb<br>CPT: 60100, 60200, 60210,60212, 60220,60225, 60260, 60240, 60252, 60280, 60281,60270, 60271
Colectomy	ICD9: 45.7,45.71, 45.72, 45.73, 45.74, 45.75, 45.76, 45.79, 45.8, 45.82, 45.83, 45.94, 17.3, 17.31, 17.32, 17.33, 17.34, 17.35, 17.36, 17.39, 45.81, 48.6, 48.61, 48.62, 48.63, 48.64, 48.65, 48.69, 48.51, 48.4, 48.40, 48.41, 48.42, 48.43, 48.49, 48.5, 48.50, 48.52, 48.59<br>ICD10: 0DBE0ZZ,0DBE3ZZ,0DBE7ZZ,0DBE8ZZ,0DBF0ZZ,0DBF3ZZ,0DBF7ZZ,0DBF8ZZ,0DBG0ZZ, 0DBG3ZZ, 0DBG7ZZ,0DBG8ZZ,0DBK0ZZ,0DBK3ZZ,0DBK7ZZ, 0DBK8ZZ,0DBL0ZZ,0DBL3ZZ, 0DBL7ZZ, 0DBL8ZZ, DBLFZZ,0DBM0ZZ,0DBM3ZZ,0DBM7ZZ,0DBM8ZZ,0DBMFZZ,0DBN0ZZ, 0DBN3ZZ, 0DBN7ZZ,0DBN8ZZ, DBNFZZ,0DTE0ZZ,0DTE7ZZ,0DTE8ZZ,0DTF0ZZ,0DTF7ZZ, 0DTF8ZZ, 0DTG0ZZ,0DTG7ZZ, 0DTG8ZZ, 0DTGFZZ,0DTK0ZZ,0DTK7ZZ,0DTK8ZZ,0DTL0ZZ, 0DTL7ZZ, 0DTL8ZZ,0DTLFZZ,0DTM0ZZ, 0DTM7ZZ, 0DTM8ZZ,0DTMFZZ,0DTN0ZZ,0DTN7ZZ, 0DTN8ZZ, 0DTNFZZ,0DBE4ZZ,0DBF4ZZ,0DBG4ZZ, 0DBK4ZZ, 0DBL4ZZ,0DBM4ZZ,0DBN4ZZ, 0DTE4ZZ, 0DTF4ZZ,0DTG4ZZ,0DTK4ZZ,0DTL4ZZ,0DTM4ZZ, 0DTN4ZZ, 0P0ZZ,0DBP3ZZ, 0DBP7ZZ, 0DBP8ZZ,0DBQ0ZZ, 0DBQ3ZZ,0DBQ7ZZ,0DBQ8ZZ,0DBQXZZ, 0DBR0ZZ, 0DBR3ZZ, 0DTP0ZZ, 0DTP7ZZ,0DTP8ZZ,0DTQ0ZZ,0DTQ7ZZ,0DTQ8ZZ,0DTR0ZZ,0DTR7ZZ, 0DTR8ZZ, 0DBP4ZZ, 0DBQ4ZZ,0DBR4ZZ,0DTP4ZZ,0DTQ4ZZ,0DTR4ZZ,0DBP0ZZ, 0DBP3ZZ,0DBP7ZZ, 0DBP8ZZ,0DTP0ZZ,0DTP7ZZ,0DTP8ZZ<br>CPT: 44140, 44141, 44143, 44144, 44145, 44146, 44147, 44150, 44151, 44153, 44155, 44156, 44157, 44158, 44160, 44204, 44205, 44206, 44207, 44208, 44210, 44211, 44212, 44213, 45113, 45114, 45116, 45123, 45395, 45397, 45402, 45110, 45111, 45112, 45119, 45120, 45121, 45123, 45111, 45160, 45717, 45172, 45190, 45113, 45114, 45116, 45126
Total Knee Arthroplasty	ICD9: 81.54<br>ICD10: 0SRC06Z,0SRC07Z,0SRC0JZ,0SRC0KZ,0SRC0LZ,0SRD06Z,0SRD07Z,0SRD0JZ, 0SRD0KZ,0SRD0LZ,0SRT07Z,0SRT0JZ,0SRT0KZ,0SRU07Z,0SRU0JZ,0SRU0KZ, 0SRU07Z,0SRU0JZ,0SRU0KZ,0SRV07Z,0SRV0JZ,0SRV0KZ,0SRW07Z<br>CPT: 27440,27441,27442,27443,27445,27446,27447
Hip Arthroplasty	ICD9: 81.51<br>ICD10: 0SR90J9,0SR90JA,0SR90JZ,0SRB0J9,0SRB0JA,0SRB0JZ<br>CPT: 27130,27132,27134,27137,27138
Laparoscopic appendectomy	ICD9: 47.01,47.11<br>ICD10: 0DBJ4b, 0DBJ8b, 0DTJ4b, 0DTJ8b<br>CPT: 44970, 44979
Laparoscopic cholecystectomy	ICD9: 51.23,51.24<br>ICD10: 0F544b, 0F548b, 0FB44b, 0FB48b, 0FC44b, 0FC48b, 0FT44b<br>CPT: 47562, 47563, 47564
Lung lobectomy	ICD9: 32.59, 32.29, 32.39, 32.49, 32.20, 32.50, 32.30, 32.41<br>ICD10: 0BBK0,0BBK0Z,0BBK0ZX,0BBK0ZZ,0BBK3,0BBK3Z,0BBK3ZX,0BBK4Z,0BBK7,0BBK7Z, 0BBK7ZZ,0BBK8,0BBK8Z,0BBL0Z,0BBL0ZX,0BBL0ZZ,0BBL3,0BBL3Z,0BBL3ZX,0BBL3ZZ, 0BBL7,0BBL7Z,0BBL7ZX,0BBL7ZZ,0BBL8,0BBL8Z,0BBL8ZX,0BBL8ZZ,0BDM,0BDM4, 0BDM4Z,0BDM4ZX,0BTK,0BTK0,0BTK0Z,0BTK0ZZ,0BTL,0BTL0,0BTL0Z,0BTL0ZZ,

© 2021 Wright JD et al. *JAMA Network Open.*

	0BTM,0BTM0,0BTM0Z,0BTM0ZZ,0BBK3ZZ,0BBK4,0BBK4ZZ,0BBK4ZX,0BBK7ZX,0BBK8ZX, 0BBK8ZZ, 0BBL4,0BBL4Z,0BBL4ZX,0BBL4ZZ,0BBL8,0BBL8ZX,0BBL8ZZ,0BDM8,0BDM8Z, 0BDM8ZX, 0BTK4,0BTK4Z,0BTK4ZZ,0BTL4,0BTL4Z,0BTL4ZZ,0BTM4,0BTM4Z,0BTM4ZZ<br>CPT: 32100, 32110, 32124, 32140, 32141, 32150, 32610, 32440, 32442, 32445, 32480, 32482, 32484, 32486, 32488, 32491, 32500, 32503, 32505, 32506, 32507, 32540, 32657, 32663, 32666, 32667, 32668, 32669, 32670, 32671, 32672, 32673, 32504, 32661, 32662, 32674
Cataract surgery	ICD9: 13.1,13.19,13.2,13.3,13.5,13.51,13.59,13.4,13.41,13.43,14.73<br>ICD10: 08DJ3ZZ,08DK3ZZ,08B43ZZ,08B53ZZ,08T43ZZ,08T53ZZ<br>CPT: 66840,66850,66920,66930,66940,66982,66983,66984
Hemorrhoidectomy	ICD9: 49.43, 49.44, 49.45, 49.46, 49.49<br>ICD10: 065Yb, 06BYb, 06LYb<br>CPT: 46250,46255,46257,46258,46260,46261,46262,46945,46946,46930,46083
Carpal tunnel surgery	ICD9: 04.43<br>ICD10: 01N50ZZ, 01N53ZZ, 01N54ZZ<br>CPT: 64721,29848
Prostatectomy	ICD9: 60, 60.1,60.11,60.12,60.13,60.14,60.15,60.18,60.19,60.2,60.21,60.29,60.3, 60.4,60.5,60.6,60.61, 60.62,60.69<br>ICD10: 0V9000Z, 0V9070Z, 0V907ZZ, 0V9080Z, 0V908ZZ, 0VC00ZZ, 0VC03ZZ, 0VC04ZZ, 0VC07ZZ, 0VC08ZZ, 0VJ40ZZ, 0VJ44ZZ, 0VP40YZ, 0VP43YZ, 0VP44YZ, 0VW40YZ, 0VW43YZ, 0VW44YZ, 0VW47YZ, 0VW48YZ, 0V507ZZ, 0V508ZZ, 0VT00ZZ, 0VT00ZZ, 0VT00ZZ, 0VT04ZZ, 0VT07ZZ, 0VT08ZZ, 0VT30ZZ, 0VT34ZZ, 0VB00ZZ, 0VB03ZZ, 0VB04ZZ, 0V500ZZ, 0V503ZZ, 0V504ZZ, 0VT00ZZ, 0VT04ZZ, 0VT07ZZ, 0VT08ZZ<br>CPT: 55831,55840,55842,55845,55866
Insomnia	ICD9: 780.52<br>ICD10: G4700b
Anxiety	ICD9: 300, 300.0, 300.00, 300.01, 300.02, 300.09, 300.1, 300.10, 300.11, 300.12, 300.13, 300.14, 300.15, 300.16, 300.19, 300.2, 300.20, 300.21, 300.22, 300.23, 300.29, 300.3, 300.4, 300.5, 300.6, 300.7, 300.8, 300.81, 300.82, 300.89, 300.9, 293.84<br>ICD10: F40b, F41b, F4322b, F4323b, F064b
Depression	ICD9: 296.2, 296.20, 296.21, 296.22, 29623, 296.24, 296.25, 296.26, 298.0, 309.1, 309.0, 309.1,311<br>ICD10: F32b, F33b, F4321b, F0632b
Substance use disorder	ICD9: 291, 292, 303, 304, 305<br>ICD10: F10b, F11b, F12b, F13b, F14b, F15b, F16b, F17b, F18b, F19b

© 2021 Wright JD et al. *JAMA Network Open.*

eTable 2. Individual Benzodiazepine Drugs Used Perioperatively and Persistently

	**Perioperative use (N=63,931)**	**Persistent use (N=12,468)**
	**N(%)**	**N(%)**
Diazepam	20,661(32.3)	2,005(16.3)
Alprazolam	18,794(29.4)	4,619(37.5)
Lorazepam	15,322(24)	3,383(27.4)
Temazepam	3,806(6)	892(7.2)
Clonazepam	3,634(5.7)	1,837(14.9)
Midazolam	1,378(2.2)	63(0.51)
Chlordiaze	1,135(1.8)	198(1.61)
Triazolam	4,83(0.8)	103(0.8)
Clorazepat	197(0.3)	59(0.5)
Flurazepam	186(0.3)	22(0.2)
Oxazepam	85(0.1)	24(0.2)
Estazolam	22(0.03)	10(0.1)
Clobazam	2(0)	0(0)
Quazepam	1(0)	0(0)

Patients may have been prescribed multiple benzodiazepines during study period.

© 2021 Wright JD et al. *JAMA Network Open.*

eTable 3. Factors Associated With at Least 30 Days' Benzodiazepine Supply During 90 to 180 Days After Operation

	Benzos supply day ≥30		*P-values*	aRR (95%CI)
	No	Yes		
	N(%)	N(%)		
**All**	55,674(87.1)	82,57(12.9)		
**Surgery type**			<.001	
Hysterectomy	10,960(87.4)	1,586(12.6)		0.81 (0.75 - 0.87)[a]
Colectomy	3,358(82.7)	701(17.3)		0.93 (0.84 - 1.03)
Knee arthroplasty	8,134(90.3)	871(9.7)		0.64 (0.58 - 0.71)[a]
Hip arthroplasty	4,241(91.1)	413(8.9)		0.60 (0.53 - 0.67)[a]
Pulmonary lobectomy	1,443(78.9)	387(21.2)		0.99 (0.87 - 1.12)
Prostatectomy	1,309(91.2)	126(8.8)		0.61 (0.50 - 0.74)[a]
Cholecystectomy	8,217(84.2)	1,547(15.8)		Referent
Thyroidectomy	2,559(87.7)	359(12.3)		0.82 (0.73 - 0.92)[b]
Appendectomy	1,528(85.7)	256(14.4)		0.98 (0.86 - 1.12)
Cataract surgery	6,100(84.4)	1,130(15.6)		1.01 (0.92 - 1.12)
Hemorrhoidectomy	5,157(93.2)	379(6.9)		0.55 (0.49 - 0.62)[a]
Carpal tunnel surgery	2,668(84.2)	502(15.8)		1.01 (0.91 - 1.12)
**Age at surgery**			<.001	
<40	9,936(86.3)	1,581(13.7)		0.98 (0.91 - 1.05)
40 -49	12,107(87.9)	1,675(12.2)		Referent
50 -59	16,457(87.4)	2,364(12.6)		1.06 (1.00 - 1.14)
60 -64	9,480(87.5)	1,360(12.6)		1.10 (1.01 - 1.18)[b]
65 -69	2,931(87.5)	418(12.5)		1.15 (1.02 - 1.28)[b]
≥70	4,763(84.7)	859(15.3)		1.35 (1.23 - 1.49)[a]
**Health insurance**			<.001	
Commercial	51,011(87.8)	7,111(12.2)		Referent
Medicaid	4,663(80.3)	1,146(19.7)		1.33 (1.02 - 1.74)[b]
**Gender**			<.001	
Male	17,394(88.3)	2,297(11.7)		Referent
Female	38,280(86.5)	5,960(13.5)		1.04 (0.98 - 1.10)
**Metropolitan statistical area (MSA)**			<.001	
MSA	8,640(87.2)	1,274(12.9)		1.00 (0.94 - 1.07)
Non -MSA	41,554(87.9)	5,731(12.1)		Referent
Unknown	5,480(81.4)	1,252(18.6)		1.10 (0.82 - 1.49)
**Region**			<.001	
Northeast	8,185(87.9)	1,123(12.1)		Referent
North Central	12,769(88.3)	1,699(11.7)		0.99 (0.92 - 1.07)
South	20,078(86.5)	3,146(13.6)		1.17 (1.09 - 1.25)[a]
West	9,550(89.8)	1,085(10.2)		0.89 (0.82 - 0.97)[b]
Unknown	5,092(80.9)	1,204(19.1)		0.89 (0.60 - 1.31)
**Year of index procedure**			<.001	
2009	7,225(86.9)	1,094(13.2)		Referent
2010	6,572(86.2)	1,050(13.8)		1.01 (0.92 - 1.10)
2011	6,617(85.6)	1,115(14.4)		1.06 (0.97 - 1.15)
2012	8,268(86.8)	1,257(13.2)		0.92 (0.85 - 1.00)[b]
2013	7,031(86.0)	1,141(14.0)		0.93 (0.85 - 1.01)
2014	6,249(87.7)	878(12.3)		0.80 (0.73 - 0.87)[a]
2015	5,724(88.3)	760(11.7)		0.71 (0.64 - 0.78)[a]
2016	5,408(88.9)	673(11.1)		0.66 (0.59 - 0.72)[a]
2017	2,580(89.9)	289(10.1)		0.58 (0.50 - 0.67)[a]
**Hospital setting**			0.40	
Outpatient	29,413(87.0)	4,403(13.0)		Referent
Inpatient	26,261(87.2)	3,854(12.8)		1.08 (1.01 - 1.15)[b]
**Elixhauser comorbidity score**			<.001	
0	35,841(88.6)	4,592(11.4)		Referent

© 2021 Wright JD et al. *JAMA Network Open.*

1	11,843(86.0)	1,929(14.0)		1.08 (1.02 - 1.14)[b]
2	4,932(83.8)	954(16.2)		1.14 (1.06 - 1.23)[b]
≥3	3,058(79.6)	782(20.4)		1.21 (1.11 - 1.31)[a]
**Insomnia**			<.001	
No	53,445(87.6)	7,551(12.4)		Referent
Yes	2,229(76.0)	706(24.1)		1.56 (1.44 - 1.69)[a]
**Anxiety**			<.001	
No	46,908(88.7)	5,975(11.3)		Referent
Yes	8,766(79.3)	2,282(20.7)		1.55 (1.47 - 1.64)[a]
**Depression**			<.001	
No	50,146(88.1)	6,782(11.9)		Referent
Yes	5,528(78.9)	1,475(21.1)		1.29 (1.21 - 1.38)[a]
**Substance use disorders**			<.001	
No	50,236(88.0)	6,873(12.0)		Referent
Yes	5,438(79.7)	1,384(20.3)		1.25 (1.17 - 1.33)[a]
**Malignant diseases**			<.001	
No	40,315(87.8)	5,591(12.2)		Referent
Yes	15,359(85.2)	2,666(14.8)		0.99 (0.93 - 1.05)
**Chemotherapy**			<.001	
No	52,299(87.8)	7,250(12.2)		Referent
Yes	3,375(77.0)	1,007(23.0)		1.48 (1.35 - 1.62)[a]
**Radiation**			<.001	
No	53,876(87.5)	7,679(12.5)		Referent
Yes	1,798(75.7)	578(24.3)		1.33 (1.19 - 1.48)[a]
**Pre-surgery opioid use**			<.001	
No	29,822(89.1)	3,668(11.0)		Referent
Yes	25,852(84.9)	4,589(15.1)		1.45 (1.38 - 1.52)a
**Perioperative opioid use**			0.50	
No	9,008(85.9)	1,479(14.1)		Referent
Yes	46,666(87.3)	6,778(12.7)		0.87 (0.81 - 0.94)b
**Persistent opioid use**			<.001	
No	52,083(87.6)	7,353(12.4)		Referent
Yes	3,591(79.9)	904(20.1)		1.99 (1.84 - 2.15)[a]
**Initial perioperative benzodiazepine use**				
**Total oral lorazepam equivalents (mg)** [1]			<.001	
<6.0	14,087(96.9)	452(3.1)		Referent
6.0 -14.9	10,856(94.1)	681(5.9)		2.16 (1.92 - 2.44)[a]
15 -29.9	15,762(88.9)	1,961(11.1)		4.04 (3.64 - 4.48)[a]
≥30	14,969(74.4)	5,163(25.7)		7.98 (7.24 - 8.80)[a]
**Daily oral lorazepam equivalents (mg)** [c]			<.001	
<1.0	12,734(88.5)	1,656(11.5)		Referent
1.0 -1.49	12,659(86.3)	2,017(13.7)		1.16 (1.08 - 1.24)[a]
1.5 -1.99	10,281(89.0)	1,275(11.0)		1.00 (0.93 - 1.08)
≥2.0	20,000(85.8)	3,309(14.2)		1.21 (1.14 - 1.28)[a]
**Total day supply**[c]			<.001	
<5	13,787(97.2)	400(2.8)		Referent
5 -9	11,898(94.8)	655(5.2)		2.05 (1.81 - 2.32)[a]
10 -22	18,993(90.3)	2,048(9.7)		3.68 (3.30 - 4.10)[a]
≥23	10,996(68.1)	5,154(31.9)		10.53 (9.50 - 11.67)[a]
**Prescription number**[c]			<.001	
1	50,567(88.8)	6,375(11.2)		Referent
≥2	5,107(73.1)	1,882(26.9)		2.12 (2.02 - 2.24)[a]
**Pre-surgery use within 30 days**[c]			<.001	
No	25,982(90.1)	2,861(9.9)		Referent
Yes	29,692(84.6)	5,396(15.4)		1.29 (1.23 - 1.36)[a]

Log -linear models with Poisson distribution and log link function. [a]P<0.0001; [b]P<0.05.

© 2021 Wright JD et al. *JAMA Network Open.*

[c]Due to the multicollinearity between perioperative benzodiazepine prescription measurements, the association between perioperative benzodiazepine use and persistent use were examined in separate models. Each model adjusted for patients' demographics and clinical factors.

© 2021 Wright JD et al. *JAMA Network Open.*

eTable 4. Factors Associated With 2 or More Benzodiazepine Prescriptions Filled 90 to 180 Days After Surgery

	Benzodiazepine prescriptions ≥2		*P*-values	aRR (95%CI)
	No	Yes		
	N(%)	N(%)		
**All**	58,476(91.5)	5,455(8.5)		
**Surgery type**			<.001	
Hysterectomy	11,364(90.6)	1,182(9.4)		0.87 (0.80 - 0.95)[b]
Colectomy	3,529(86.9)	530(13.1)		0.96 (0.85 - 1.08)
Knee arthroplasty	8,474(94.1)	531(5.9)		0.60 (0.53 - 0.68)[a]
Hip arthroplasty	4,402(94.6)	252(5.4)		0.55 (0.47 - 0.64)[a]
Pulmonary lobectomy	1,543(84.3)	287(15.7)		0.97 (0.84 - 1.13)
Prostatectomy	1,360(94.8)	75(5.2)		0.55 (0.43 - 0.71)[a]
Cholecystectomy	8,700(89.1)	1,064(10.9)		Referent
Thyroidectomy	2,719(93.2)	199(6.8)		0.71 (0.61 - 0.83)[a]
Appendectomy	1,612(90.4)	172(9.6)		0.95 (0.81 - 1.11)
Cataract surgery	6,643(91.9)	587(8.1)		0.97 (0.85 - 1.10)
Hemorrhoidectomy	5,285(95.5)	251(4.5)		0.56 (0.49 - 0.64)[a]
Carpal tunnel surgery	2,845(89.8)	325(10.3)		0.98 (0.87 - 1.12)
**Age at surgery**			<.001	
<40	10,323(89.6)	1,194(10.4)		1.05 (0.97 - 1.14)
40 -49	12,630(91.6)	1,152(8.4)		Referent
50 -59	17,230(91.6)	1,591(8.5)		1.06 (0.98 - 1.15)
60 -64	9,978(92.1)	862(8)		1.06 (0.96 - 1.16)
65 -69	3,125(93.3)	224(6.7)		0.98 (0.85 - 1.14)
≥70	5,190(92.3)	432(7.7)		1.14 (1.00 - 1.29)[b]
**Health insurance**			<.001	
Commercial	53,587(92.2)	4,535(7.8)		Referent
Medicaid	4,889(84.2)	920(15.8)		1.42 (1.04 - 1.96)[b]
**Gender**			<.001	
Male	18,164(92.3)	1,527(7.8)		Referent
Female	40,312(91.1)	3,928(8.9)		0.97 (0.91 - 1.04)
**Metropolitan statistical area (MSA)**			<.001	
MSA	9,074(91.5)	840(8.5)		1.04 (0.96 - 1.12)
Non -MSA	43,660(92.3)	3,625(7.7)		Referent
Unknown	5,742(85.3)	990(14.7)		1.20 (0.82 - 1.76)
**Region**			<.001	
Northeast	8,695(93.4)	613(6.6)		Referent
North Central	13,394(92.6)	1,074(7.4)		1.13 (1.02 - 1.25)[b]
South	21,138(91.0)	2,086(9.0)		1.37 (1.25 - 1.50)[a]
West	9,914(93.2)	721(6.8)		1.05 (0.94 - 1.17)
Unknown	5,335(84.7)	961(15.3)		1.01 (0.63 - 1.64)
**Year of index procedure**			<.001	
2009	7,586(91.2)	733(8.8)		Referent
2010	6,884(90.3)	738(9.7)		1.02 (0.92 - 1.14)
2011	7,007(90.6)	725(9.4)		1.00 (0.90 - 1.11)
2012	8,716(91.5)	809(8.5)		0.91 (0.82 - 1.01)
2013	7,436(91.0)	736(9.0)		0.90 (0.81 - 1.00)
2014	6,554(92.0)	573(8.0)		0.77 (0.69 - 0.86)[a]
2015	5,972(92.1)	512(7.9)		0.69 (0.61 - 0.77)[a]
2016	5,641(92.8)	440(7.2)		0.61 (0.54 - 0.69)[a]

© 2021 Wright JD et al. *JAMA Network Open.*

2017	2,680(93.4)	189(6.6)		0.54 (0.45 - 0.64)[a]
**Hospital setting**			<.001	
Outpatient	31,051(91.8)	2,765(8.2)		Referent
Inpatient	27,425(91.1)	2,690(8.9)		1.14 (1.05 - 1.23)[b]
**Elixhauser comorbidity score**			<.001	
0	37,405(92.5)	3,028(7.5)		Referent
1	12,504(90.8)	1,268(9.2)		1.06 (1.00 - 1.14)
2	5,253(89.3)	633(10.8)		1.12 (1.03 - 1.23)[b]
≥3	3,314(86.3)	526(13.7)		1.15 (1.04 - 1.27)[b]
**Insomnia**			<0.01	
No	56,019(91.8)	4,977(8.2)		Referent
Yes	2,457(83.7)	478(16.3)		1.54 (1.40 - 1.70)[a]
**Anxiety**			<.001	
No	49,007(92.7)	3,876(7.3)		Referent
Yes	9,469(85.7)	1,579(14.3)		1.57 (1.47 - 1.68)[a]
**Depression**			<.001	
No	52,520(92.3)	4,408(7.7)		Referent
Yes	5,956(85.1)	1,047(15)		1.33 (1.23 - 1.43)[a]
**Substance use disorders**			<.001	
No	52,729(92.3)	4,380(7.7)		Referent
Yes	5,747(84.2)	1,075(15.8)		1.36 (1.26 - 1.47)[a]
**Malignant diseases**			<.001	
No	42,290(92.1)	3,616(7.9)		Referent
Yes	16,186(89.8)	1,839(10.2)		0.97 (0.91 - 1.04)
**Chemotherapy**			<.001	
No	54,942(92.3)	4,607(7.7)		Referent
Yes	3,534(80.7)	848(19.4)		1.83 (1.65 - 2.03)[a]
**Radiation**			<.001	
No	56,600(92.0)	4,955(8.1)		Referent
Yes	1,876(79.0)	500(21.0)		1.48 (1.31 - 1.68)[a]
**Pre-surgery opioid use**			<.001	
No	31,206(93.2)	2,284(6.8)		Referent
Yes	27,270(89.6)	3,171(10.4)		1.61 (1.51 - 1.72)[a]
**Perioperative opioid use**			0.83	
No	9,711(92.6)	776(7.4)		Referent
Yes	48,765(91.3)	4,679(8.8)		0.95 (0.86 - 1.04)
**Persistent opioid use**			<.001	
No	54,665(92.0)	4,771(8.0)		Referent
Yes	3,811(84.8)	684(15.2)		2.29 (2.08 - 2.51)[a]
**Initial perioperative benzodiazepine use**				
**Total oral lorazepam equivalents (mg)** [c]			<.001	
<6.0	14,179(97.5)	360(2.5)		Referent
6.0 -14.9	11,062(95.9)	475(4.1)		1.84 (1.60 - 2.11)[a]
15 -29.9	16,476(93.0)	1,247(7.0)		3.14 (2.78 - 3.53)[a]
≥30	16,759(83.3)	3,373(16.8)		6.17 (5.52 - 6.89)[a]
**Daily oral lorazepam equivalents (mg)** [c]			<.001	
<1.0	13,475(93.6)	915(6.4)		Referent

© 2021 Wright JD et al. *JAMA Network Open.*

1.0 -1.49	13,477(91.8)	1,199(8.2)		1.25 (1.15 - 1.37)[a]
1.5 -1.99	10,706(92.6)	850(7.4)		1.18 (1.07 - 1.30)[b]
≥2.0	20,818(89.3)	2,491(10.7)		1.57 (1.45 - 1.69)[a]
**Total day supply** [c]			<.001	
<5	13,846(97.6)	341(2.4)		Referent
5 -9	11,969(95.4)	584(4.7)		2.11 (1.84 - 2.41)[a]
10 -22	19,447(92.4)	1,594(7.6)		3.27 (2.91 - 3.69)[a]
≥23	13,214(81.8)	2,936(18.2)		6.84 (6.11 - 7.67)[a]
**Prescription number** [c]			<.001	
1	52,979(93.0)	3,963(7.0)		Referent
≥2	5,497(78.7)	1,492(21.4)		2.57 (2.41 - 2.73)[a]
**Pre-surgery use within 30 days** [c]			<.001	
No	26,820(93.0)	2,023(7.0)		Referent
Yes	31,656(90.2)	3,432(9.8)		1.19 (1.12 - 1.26)[a]

Log -linear models with Poisson distribution and the link function. [a] P<0.0001; [b] P<0.05.

[c] Due to the multicollinearity between perioperative benzodiazepine prescription measurements, the association between perioperative benzodiazepine use and persistent use were examined in separate models. Each model adjusted for patients' demographics and clinical factors.

© 2021 Wright JD et al. *JAMA Network Open.*
